# Diagnosis of complicated FEVR preoperatively and intra−/post-operatively: characteristics and risk factors for diagnostic timing

**DOI:** 10.1186/s12886-019-1128-8

**Published:** 2019-06-08

**Authors:** Fengjie Xia, Jiao Lyu, Ping Fei, Peiquan Zhao

**Affiliations:** Department of Ophthalmology, Xinhua Hospital, Affiliated to Medicine School of Shanghai Jiaotong University, No. 1665, Kongjiang Road, Shanghai, 200092 China

**Keywords:** Familial exudative vitreoretinopathy, Diagnosis timing, Risk factor, Clinical characteristic

## Abstract

**Background:**

To delineate the characteristics of complicated familial exudative vitreoretinopathy (FEVR) patients diagnosed before surgery or intra−/post-operatively and to analyze the risk factors for the diagnostic timing.

**Methods:**

Forty-eight patients who underwent surgery and were diagnosed as FEVR in our department were retrospectively reviewed. Data were collected including the demographic and clinical characteristics of these patients. FEVR patients were divided into 2 groups according to the diagnostic timing: FEVR diagnosed pre-operatively (23 patients), FEVR diagnosed intra−/post-operatively (25 patients). Multivariable analysis was applied for analyzing the risk factors for diagnostic timing.

**Results:**

The clinical characteristics of the FEVR patients were of great variability, including retinal detachment (RD), disappear of anterior chamber, retrolental membrane, epiretinal membrane (ERM), vitreous hemorrhage (VH), myopic foveoschisis (MF), lamellar macular hole (LMH), high myopia (HM). And the referral diagnosis or pre-operative diagnosis were always non-specific. The majority of the referral or preoperative diagnosis were unilateral RD (52.1%), bilateral RD (8.3%), unilateral persistent fetal vasculature (PFV) (8.3%), bilateral PFV (4.2%). There are two risk factors for the complicated FEVR cases diagnosed as FEVR preoperatively: pre-operative ocular manifestations with RD only (OR, 0.104; *p*-value, 0.022), positive parent’s fluorescein angiography (FA) (OR, 0.105; *p*-value, 0.035).

**Conclusions:**

The phenotypes of FEVR were greatly variable, they can mimic many non-specific vitreoretinal disorders. The most non-specific referral diagnosis/pre-operative diagnosis was unilateral RD, bilateral RD, unilateral PFV, bilateral PFV. A positive family history or a simple ocular presentation with RD only could contribute to diagnose FEVR preoperatively.

## Background

Familial exudative vitreoretinopathy (FEVR) is an inheritable vitreoretinal disease which is caused by anomalous and incomplete retinal angiogenesis. Clinically, FEVR is characterized by avascular peripheral retina, excessive vessel branchings, increased straightening vascular branching, vitreous adherence, retinochoroidal degeneration, macular and disc dragging, retinal exudation, radial retinal folds, and exudative, and/or tractional retinal detachments (RD) [1]. The expressivity of FEVR can be asymptomatic or with greatly variable [2]. FEVR staging system, presented by Pendergast and Trese in 1998, were widely used in the clinical research [3]. The staging of bilateral eyes in the same individual can be greatly asymmetric, such as one eye being stage 1–2 with no symptom, while the contralateral eye being stage 3–5 with RD [4]. Additionally, the disease course of FEVR was greatly skipped and unpredictable. It can be stable in stage 1–2 in the lifetime or be rapidly progressive from stage 1–2 to stage 3–5. Therefore, early diagnosis of FEVR is important and it is essential for FEVR patients to undergo lifelong regular follow-up [5].

However, in the clinic, FEVR is always mistaken for non-specific vitreoretinopathies and the diagnosis of FEVR is easily missed. Ranchod et.al reported that 71.7% FEVR patients were referred to their institution with a nonspecific diagnosis [4]. Because the manifestations of FEVR eyes are of greatly diverse form, such as RD, leukocoria, disappearance of anterior chamber, VH [6, 7]. Besides, due to the peripheral retinochoroidal scars of photocoagulation or cryotherapy, the diagnosis of FEVR in patients who had prior vitreoretinal surgery would be more challenging. Since FEVR is an inherited retinopathy, missed diagnosis of FEVR is not conducive to the patient’s lifetime follow-up, and do not contribute to genetic counseling for those of child-bearing age. Many prior clinical studies have reported the clinical characteristics of untreated FEVR patients and surgery outcomes of FEVR-RD patients [4, 8–11]. However, there are few studies reported the diagnostic timing of FEVR patients who underwent surgery.

In the study, we retrospectively analyzed 48 FEVR patients who underwent surgery and diagnosed as FEVR finally from March 2010 to May 2018 in Xinhua hospital. In the cohort, the diagnostic timing of FEVR patients was variable, including preoperatively, during the operation and in the follow-up of post-operation. We collected the demographic features, clinical characteristics of the cohort and their first surgery in our department. Furtherly, we analyzed the risk factors for diagnostic timing of the complicated FEVR patients. It would contribute to ophthalmologists to diagnose FEVR much earlier and prevent missed diagnosis of FEVR.

## Methods

### Patients

The retrospective study was conducted in accordance with the tenets of the Declaration of Helsinki and was approved by the Ethics Committee of Xinhua Hospital affiliated to Shanghai Jiao Tong University School of Medicine, Shanghai, China. All patients provided an informed consent prior to participating into the study. A total of 48 patients with FEVR who underwent surgery were studied. The 48 patients were diagnosed as FEVR in our department between March 2010 and May 2018. We found that these FEVR patients had one of the following findings in at least one eye: RD, disappear or shallow of anterior chamber, retrolental membrane, leukocoria, ERM, vitreous hemorrhage (VH), myopic foveoschisis (MF). Among those ocular findings, RD is the most common ocular manifestations occurred in these FEVR patients. In the study, except RD, the rest of ocular findings as mentioned above were defined as other ocular manifestations. The clinical diagnostic criteria for FEVR were: (1) a lack of peripheral retinal vascular development, (2) full-term or preterm birth with a disease course not consistent with retinopathy of prematurity, and (3) variable degrees of non-perfusion, vitreoretinal traction, sub-retinal exudation, or retinal neovascularization occurring at any age. FEVR was classified according to the clinical staging criteria described by Pendergast and Trese. The inclusion criteria for the 48 FEVR patients in this study were as follows: (1) all patients underwent surgery in our department, (2) the diagnosis of FEVR was confirmed by careful peripheral fundus examination, positive findings in patient FA or positive family history. Patients were excluded if the diagnosis of FEVR was questionable (i.e., Unilateral FEVR that have negative genetic sequencing and no family history were excluded). Patients were excluded if FEVR cases did not undergo surgery.

Data collected from charts included gender, age presenting to our outpatient clinic, clinical presentation in each eye (stage, other ocular findings except RD), therapy procedure, referral diagnosis (if provided), preoperative diagnosis, family history, prior ocular surgery, premature birth, ocular trauma, ultra-widefield fundus image, diagnostic timing, year of initial clinic visit, year of FEVR diagnosis, patient FA. Patient FA and their parents FA were performed when available. There were some patients and parents did not undergo FA, because they were too young to cooperate with FA or were sensitive to fluorescein sodium.

According to the diagnostic timing, the 48 FEVR patients were divided into 2 groups: group 1, FEVR diagnosed preoperatively; group 2, FEVR diagnosed intra−/post-operatively. Patients, who were clearly identified with avascular peripheral retina and vascular anomalies consistent with FEVR before vitreoretinal or scleral buckling surgery in our department, were classified as FEVR diagnosed preoperatively. Patients, who had no evidence associated with FEVR preoperatively and were diagnosed as FEVR through intraoperative FA or impressing peripheral sclera during surgery, were classified as FEVR diagnosed intraoperatively. Patients, who were identified with avascular peripheral retina and vascular anomalies consistent with FEVR during postoperative follow-up, were classified as FEVR diagnosed postoperatively.

### Statistical analysis

The data were analyzed using SPSS 24.0 software (SPSS, Inc., Chicago, IL, USA). Descriptive summary statistics were presented as mean and SD, as range and median for continuous variables, and as number and proportion for categorical data. The differences between the means were tested using t-test. The differences between the groups were tested using Chi-square test or Mann-Whitney U test. Risk factors for diagnostic timing were analyzed using binary logistic regression. Potential risk factors obtained from clinical charts were the patient’s age at initial clinic visit (years), gender, preterm birth, ocular trauma, surgery in other hospital before first clinic visit, preoperative ocular manifestations, ocular involvement, clinical stage of patients at initial presentation, clinical stage in contralateral eye, year of diagnosis, year at initial clinic visit (years), wide-angle fundus imaging preoperatively, initial surgery in our hospital, father/mother’s FA. These clinical factors were included in univariate analysis. A logistic multivariate regression forward stepwise model was constructed to identify independent risk factors from variables demonstrating statistically significant associations (*P* < 0.05) with risks of FEVR diagnosis preoperatively by univariate analysis.

## Results and case descriptions

### Subjects

Patient demographics are shown in Table 1. Thirteen females and 35 males were included in the study. The age of patients ranged from 3 months to 58 years and the median was 12 years. 47.9% of cases were diagnosed as FEVR preoperatively, while 52.1% cases were diagnosed as FEVR intra−/post-operatively. In the study, the stage of FEVR patient was defined as the stage of more severe eye. 95.8% of patients were advanced FEVR (stage 3–5), while the contralateral eyes of 81.2% of FEVR patients were mild FEVR (stage 1–2). Moreover, 95.8% of the FEVR patients were diagnosed as non-specific retinopathy in other hospital or before surgery in our hospital, including unilateral RD (52.1%), bilateral RD (8.3%), unilateral PFV (4.2%), bilateral PFV (4.2%). In the cohort, the majority of patients were bilateral FEVR and only one patient was unilateral FEVR. The unilateral FEVR patient has a positive family history and her contralateral eye has no vascular abnormalities.

**Table 1:** Demographic, ocular characteristics, referral or preoperative diagnosis of FEVR patients

Variable	Total (*n* = 48)	FEVR diagnosed pre-operatively (*n* = 23)	FEVR diagnosed intra−/post-operatively (*n* = 25)	*P* value
Age at initial clinic visit (years)	14.22 ± 13.87	15.65 ± 10.17	12.90 ± 16.68	0.499*
Age range (years), (median)	0.25–58 (12)	0.25–41 (14)	0.25–58 (6)	
Sex				
Female	13 (27.1)	4 (17.4)	9 (36.0)	0.147#
Male	35 (72.9)	19 (82.6)	16 (64.0)	
Clinical stage of patients at initial presentation, n (%) #				
Stage 2	2 (4.2)	0 (0.0)	2 (8.0)	0.398^
Stage 3	11 (22.9)	7 (30.4)	4 (16.0)	
Stage 4	12 (25.0)	7 (30.4)	5 (20.0)	
Stage 5	23 (47.9)	9 (39.1)	14 (56.0)	
Clinical stage in contralateral eye, n (%)				
NON-FEVR	1 (2.1)	0 (0.0)	1 (4.0)	0.216^
Stage 1	22 (45.8)	10 (43.5)	12 (48.0)	
Stage 2	17 (35.4)	7 (30.4)	10 (40.0)	
Stage 3	5 (10.4)	3 (13.0)	2 (8.0)	
Stage 4	1 (2.1)	1 (4.4)	0 (0.0)	
Stage 5	2 (4.2)	2 (8.7)	0 (0.0)	
Patient FA				
+	35 (72.9)	15 (65.2)	20 (80.0)	–
N/A	13 (27.1)	8 (34.8)	5 (20.0)	
Referral diagnosis/pre-operative diagnosis, n (%)				
Unilateral RD	25 (52.1)	14 (60.9)	11 (44.0)	–
Bilateral RD	4 (8.3)	3 (13.0)	1 (4.0)	
Bilateral PFV	2 (4.2)	2 (8.7)	0 (0.0)	
Unilateral PFV	4 (8.3)	1 (4.3)	3 (12.0)	
Ocular trauma	1 (2.1)	1 (4.3)	0 (0.0)	
Bilateral FEVR	2 (4.2)	2 (8.7)	0 (0.0)	
Unilateral PFV + ROP	1 (2.1)	0 (0.0)	1 (4.0)	
Unilateral RB	2 (4.2)	0 (0.0)	2 (8.0)	
Unilateral congenital cataract + VH + RD	1 (2.1)	0 (0.0)	1 (4.0)	
Unilateral RD + VH	1 (2.1)	0 (0.0)	1 (4.0)	
VH + contralateral ERM	1 (2.1)	0 (0.0)	1 (4.0)	
Unilateral VH + aphakia	1 (2.1)	0 (0.0)	1 (4.0)	
Bilateral MF + LMH + HM	1 (2.1)	0 (0.0)	1 (4.0)	
Bilateral congenital cataract+ unilateral PFV	1 (2.1)	0 (0.0)	1 (4.0)	
Unilateral ERM	1 (2.1)	0 (0.0)	1 (4.0)	

*RD* Retinal detachment, *PFV* Persistent fetal vasculature, *ROP* Retinopathy of prematurity, *RB* Retinoblastoma, *VH* Vitreous hemorrhage, *ERM* Epiretinal membrane, *MF* Myopic foveoschisis, *LMH* Lamellar macular hole, *HM* High myopia

* t-test

# Chi-square test

^ Mann-Whitney U test

### Risk factors

A total of 14 potential factors entered the univariate analysis, and 5 were found statistically associated with diagnosis of FEVR preoperatively (Table 2). In multivariate regression analysis, two factors were identified significantly associated with diagnosis of FEVR preoperatively: preoperative ocular manifestations (OR, 0.104; P, 0.022) and father/mother’s FA (OR, 0.105; P, 0.035). The patients who had RD only before surgery and the cases whose parents’ FA were positive were more likely to be diagnosed as FEVR preoperatively. In contrary, the patients who had more complicated ocular manifestations and the cases who had no family history of FEVR were more susceptible to be diagnosed as FEVR intra−/post-operatively. Namely, these FEVR cases would be prone to be missed diagnosed preoperatively. The reasons of missed diagnosis of FEVR preoperatively were summarized in the Table 3. In the study, the most common reason for missed diagnosis of FEVR preoperatively was that there was no ultra-wide-field scanning laser ophthalmoscopy at initial clinic visit or preoperatively.

**Table 2:** Univariate and Multivariate Logistic Regression Analysis of Risk Factors of diagnostic timing in FEVR patients

Risk factors	FEVR diagnosed pre-operatively (*n* = 23)	FEVR diagnosed intra−/post-operatively (*n* = 25)	Univariate Analysis		Multivariate Analysis	
			OR (95% CI)	*P*	OR (95% CI)	*P*
Preoperative ocular manifestations, n (%) (no. of patients)						
RD only	19 (82.6)	8 (32.0)	0.099 (0.025–0.389)	0.001	0.104 (0.015–0.725)	0.022
Other manifestations except/without RD	4 (17.4)	17 (68.0)				
Father/mother FA, n (%)						
-	2 (8.7)	9 (36.0)	0.141 (0.023–0.857)	0.033	0.105 (0.013–0.855)	0.035
+	11 (47.8)	7 (28.0)				
N/A	10 (43.5)	9 (36.0)				
Year of diagnosis, n (%)						
2010–2015	7 (30.4)	17 (68.0)	0.161 (0.044–0.583)	0.005		
2016–2018	16 (69.6)	8 (32.0)				
Year at initial clinic visit (years), n (%)						
2007–2014	7 (30.4)	19 (76.0)	0.138 (0.039–0.496)	0.002		
2015–2018	16 (69.6)	6 (24.0)				
Wide-angle fundus imaging preoperatively, n (%)						
No	6 (26.1)	20 (80.0)	0.088 (0.023–0.341)	0.000		
Yes	17 (73.9)	5 (20.0)				
Ocular trauma, n (%)						
No	17 (73.9)	24 (96.0)	0.118 (0.013–1.072)	0.058		
Yes	6 (26.1)	1 (4.0)				
Clinical stage in contralateral eye, n (%)						
Stage 1-Stage 2	17 (73.9)	23 (92.0)	4.059 (0.728–22.637)	0.110		
Stage 3-Stage 5	6 (26.1)	2 (8.0)				
Age at initial clinic visit (years), n (%)						
0.25–12	10 (43.5)	16 (64.0)	0.433 (0.136–1.381)	0.157		
13–58	13 (56.5)	9 (36.0)				
Gender, n (%)						
Female	4 (17.4)	9 (36.0)	0.374 (0.097–1.447)	0.154		
Male	19 (82.6)	16 (64.0)				
Preterm birth, n (%)						
Yes	0 (0.0)	1 (4.0)	0.000 (0.000-)	1.000		
No	23 (100.0)	24 (96.0)				
Surgery in other hospital before first clinic visit, n (%)						
No	21 (91.3)	19 (76.0)	3.316 (0.596–18.451)	0.171		
Yes	2 (8.7)	6 (24.0)				
Ocular involvement, n (%)						
Bilateral	6 (26.1)	5 (20.0)	0.908 (0.253–3.251)	0.882		
Unilateral	17 (73.9)	20 (80.0)				
Clinical stage of patients at initial presentation, n (%)^a^						
Stage 1-Stage 4	14 (60.9)	11 (44.0)	1.980 (0.626–6.259)	0.245		
Stage 5	9 (39.1)	14 (56.0)				
Initial surgery in our hospital, n (%)						
SB/SE	7 (30.4)	5 (20.0)	1.750 (0.466–6.568)	0.407		
PPV/L	16 (69.6)	20 (80.0)				

Cut-off value based on median value in the total sample of 48 patients

^a^The clinical stage of FEVR patients were defined as the clinical stage of severer eye

**Table 3:** The reasons of missed diagnosis of FEVR preoperatively

Reasons of missed diagnosis of FEVR preoperatively	Cases (*n* = 28)^a^
Age<3 years, unilateral leukocoria with invisible fundus + contralateral fundus seemingly normal	8
Age ≥ 3 years, without ultra-wide-field scanning laser ophthalmoscopy at initial clinic visit or preoperatively	9
Bilateral vitreoretinal surgery or peripheral retinal photocoagulation in other hospital before the first clinic visit	2
Unilateral VH + contralateral phthisis bulbi	2
Unilateral VH + contralateral ERM	1
Unilateral RD + contralateral ERM	1
Unilateral ERM	1
Bilateral pathological myopia + myopic foveoschisis + lamellar macular hole	1
3 years < age <4 years, poor cooperation, ultra-wide-field scanning laser ophthalmoscopy failed to capture the peripheral retina	1
Premature birth history	1
Ocular trauma	1

^a^The number of cases in Table 3 is totally 28, which is more than the number of FEVR patients diagnosed intra−/post-operatively (*n* = 25). Because some patients had multiple reasons of missed diagnosis of FEVR preoperatively

### Case descriptions

#### Other ocular manifestations except RD at the first clinic visit in our department

Table 4 summarized the clinical characteristics of the complicated FEVR patients who had other ocular manifestation except RD at the first clinic visit. The expressivity of FEVR patients were greatly variable. The main clues for these complicated FEVR were listed in the Table 5.

**Table 4:** The clinical characteristics of FEVR patients with other ocular manifestations except RD

Patient no. (*n* = 21)	Age at initial clinic visit	Sex	Eye (*n* = 32)	FEVR stage	Ocular manifestations except RD	Operation numbers in other referral hospitals	Initial therapy in our hospital	Patient FA	Parents FA	Preoperative diagnosis	Final diagnosis	Diagnostic timing
1	3mos	F	OD	5	Corneal leucoma, AC (−), VH	0	L	N/A	+	OU FEVR	OU FEVR	Pre-op
			OS	5	Corneal leucoma, AC (−), VH		L					
2	4mos	M	OD	5	Corneal leucoma, AC (−), a cord rising from the optic head to the lens, prolonged ciliary process	0	LV	N/A	+	OU FEVR	OU FEVR	Pre-op
			OS	5	Corneal leucoma, AC (−), a cord rising from the optic head to the lens, prolonged ciliary process	0	LV					
3	10 yrs	M	OD	5	Repaired corneal laceration, hyphema	1	LV + SiO	N/A	N/A	OU FEVR	OU FEVR	Pre-op
4	4mos	M	OD	5	Corneal leucoma, AC (−), falciform retinal fold	0	LV+ FA	+	+	OU FEVR	OU FEVR	Pre-op
5	4mos	M	OD	5	Microphthalmia, retrolental membrane	0	LV + FA	+	–	OD PFV, OD ROP	OU FEVR	Intra-op
6	3mos	M	OD	5	Corneal leucoma, AC (−), lens opacity	0	LV + FA	+	–	OU suspected FEVR	OU FEVR	Intra-op
7	5mos	M	OS	5	AC (−), posterior iris adhesion	0	L + FA	+	–	OU suspected FEVR	OU FEVR	Intra-op
8	3mos	M	OD	5	Corneal leucoma, AC (−)	0	L + FA	+	N/A	OD PFV	OU FEVR	Intra-op
9	6mos	M	OD	5	Retrolental membrane	0	LV + FA	+	+	OD PFV	OU FEVR	Intra-op
10	4mos	M	OD	5	AC (−), retrolental membrane	0	L + FA	+	–	OD Rb	OU FEVR	Intra-op
11	3 yrs	F	OD	5	Cataract, VH	0	LV + FA	+	+	OD congenital cataract, VH, RD	OU FEVR	Intra-op
12	4 yrs	F	OD	3	VH	0	V	N/A	+	OD RD, OD VH, OS phthisis	OU FEVR	Intra-op
			OS	5	Phthisis	0						
13	5 yrs	F	OD	2	ERM	0						
			OS	2	VH	0	LV	N/A	+	OD ERM, OS VH	OU FEVR	Intra-op
14	58 yrs	F	OD	5	Phthisis bulbi	0						
			OS	2	VH, nystagmus	1	V	N/A	N/A	OD phthisis, OS VH, aphakia	OU FEVR	Intra-op
15	58 yrs	F	OD	1	MF, LMH, HM	0						
			OS	2	MF, LMH, HM	0	Phaco+V + ILMpeeling+C3F8	+	N/A	OU MF, LMH, PM	OU FEVR	Intra-op
16	25 yrs	M	OD	2	ERM	0	V + SiO					
			OS	4	SiO tamponade	1	SiOR+LV + SiO	N/A	–	OD ERM, OS RD, OS SiO tamponade	OU FEVR	Intra-op
17	6mos	F	OS	5	Shallow AC, anterior iris adhesion, esotropia	0	L	OS+, OD-	+	OS PFV	OS Unilateral FEVR	Post-op
18	23 yrs	F	OD	1	Peripheral laser spots, HM	0						
			OS	3	peripheral laser spots, HM	0	SB	+	–	OS RD	OU FEVR	Post-op
19	18 yrs	M	OD	4	SiO tamponade, HM	3	SiOR+V + SiO	+	N/A	OD recurrent RD	OU FEVR	Post-op
			OS	2	Retinal tear surrounded by laser spots	0						
20	40 yrs	M	OD	5	HM	1	V + SiO	+	N/A	OD RD	OU FEVR	Post-op
			OS	2	Superiotemporal retinal dry hole	1	PC					
21	8 yrs	M	OD	3	RD occurred later	0	SB				OU FEVR	Post-op
			OS	2	ERM	0	V + ILMpeeling+C3F8	+	N/A	OS ERM		

*RD* Retinal detachment, *AC (−)* Disappeared anterior chamber, *VH* Vitreous hemorrhage, *ERM* Epiretinal membrane, *MF* Myopic foveoschisis, *LMH* Lamellar macular hole, *HM* High myopia, *L* Lensectomy, *LV* Lensectomy and vitrectomy, *V* Vitrectomy, *SB* Scleral buckling, *PC* Photocoagulation, *SiO* Silicone oil, *SiOR* Remove silicone oil, *ILM* Internal limiting membrane

**Table 5:** The main clues for these complicated FEVR

Main clue for diagnosing FEVR	Cases (*n* = 21)
Complicated FEVR patients diagnosed as FEVR preoperatively	
Family history (+)	3
Avascular peripheral area and peripheral vascular anomalies in the contralateral eye	1
Complicated FEVR patients diagnosed as FEVR intraoperatively	
Intraoperative FA (+) in the contralateral eye	7
Avascular peripheral area and peripheral vascular anomalies were found when the peripheral sclera was impressed during the operation	5
Complicated FEVR patients diagnosed as FEVR postoperatively	
Radial retinal fold involved in all major retinal vessels during the follow-up	1
Peripheral avascular zone was found during the follow-up	4

#### Complicated FEVR patients diagnosed as FEVR preoperatively

**Fig. 1:**
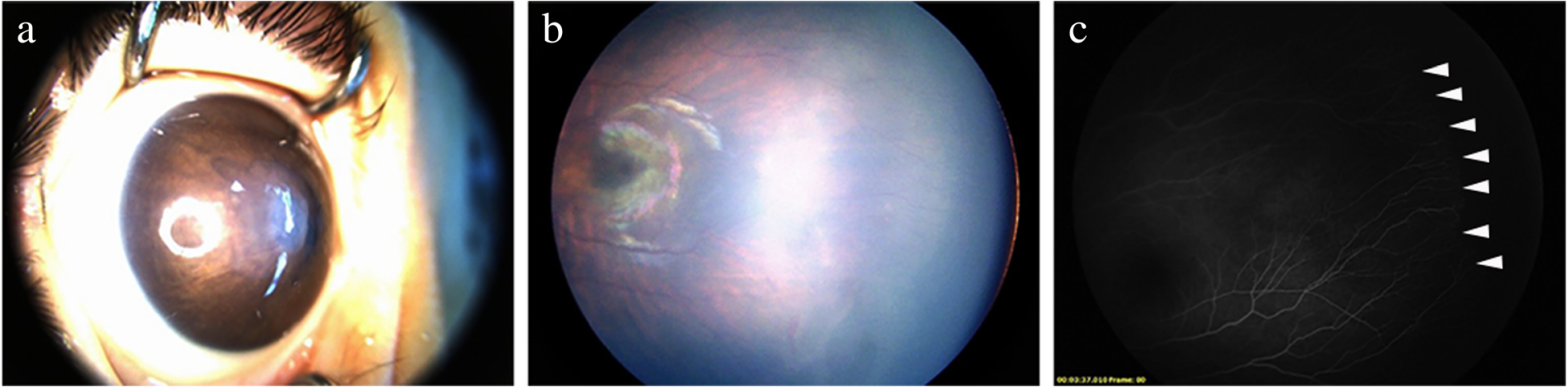
Anterior segment photograph and fundus image in patient no. 4. **a** The anterior chamber of right eye was disappeared and the central cornea was edema. The fundus of right eye was absolutely invisible. **b** The fundus image of left eye by Retcam showed that there were peripheral avascular zone and supernumerous ramification of vascular. **c** The angiogram of left eye indicated that there was avascular area in peripheral retina, increased ramification of vessel (arrowhead). The FA image confirmed the diagnosis of FEVR

In FEVR diagnosed preoperatively, positive family history was essential for FEVR patients with bilateral invisible fundus and was important for FEVR patients with unilateral invisible fundus. Patient no. 4 had only unilateral disappeared anterior chamber and positive family history, so that we diagnosed him as FEVR but not PFV. And in the operation, FA performed in the contralateral eye confirmed the diagnosis of FEVR (Fig. 1).

#### Complicated FEVR patients diagnosed as FEVR intraoperatively

**Fig. 2:**
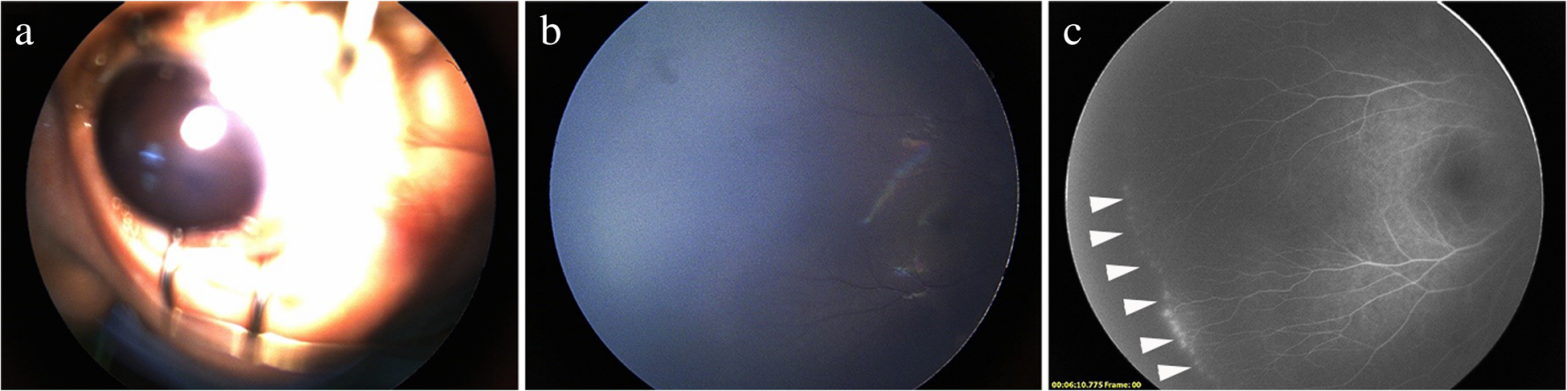
Anterior segment photograph and fundus image in patient no. 7. **a** the anterior chamber of left eye was disappeared and posterior iris was adhered. The fundus of left eye was absolutely invisible. **b** the fundus image of right eye by Retcam showed that the peripheral retina was not clear and the fundus seemed likely to be normal. **c** FA was performed during the surgery under general anesthesia. The angiogram of right eye suggested that there was avascular area, increased ramification of vessel and bulb-like or telangiectatic endings in the periphery (arrowhead)

**Fig. 3:**
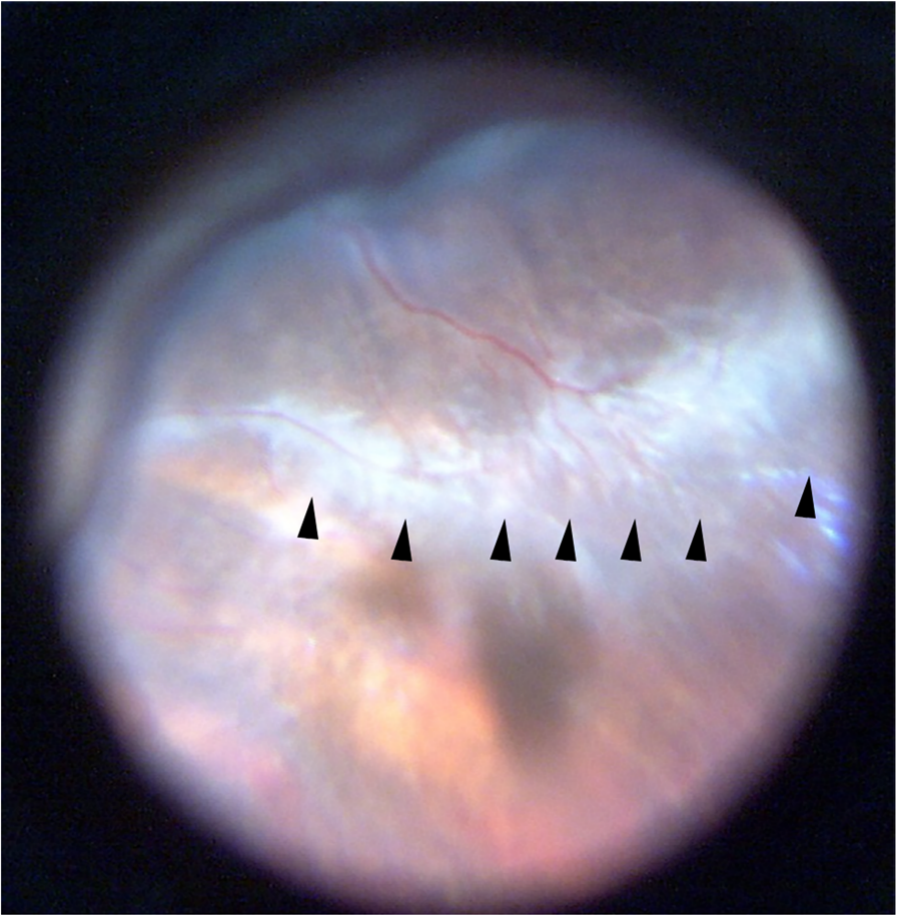
The intraoperative fundus image of the right eye in patient no.12. During the pars plana vitrectomy, the peripheral avascular zone was found when the sclera was impressed (arrowhead)

Patient no. 5–16 were FEVR cases diagnosed during the surgery. We performed intraoperative FA examination under general anesthesia in the suspected FEVR patients ≤3 years old (patient no. 5–11). These patients had only one eye involved seriously with invisible fundus, while the contralateral eye seemed like a healthy one (Fig. 2). Intraoperative FA could help us to diagnose FEVR correctly. Patient no. 12–16 were patients > 3 years old. We found avascular peripheral area and peripheral vascular anomalies when we impressed the peripheral sclera during the vitrectomy (Fig. 3).

#### Complicated FEVR patients diagnosed as FEVR postoperatively

**Fig. 4:**
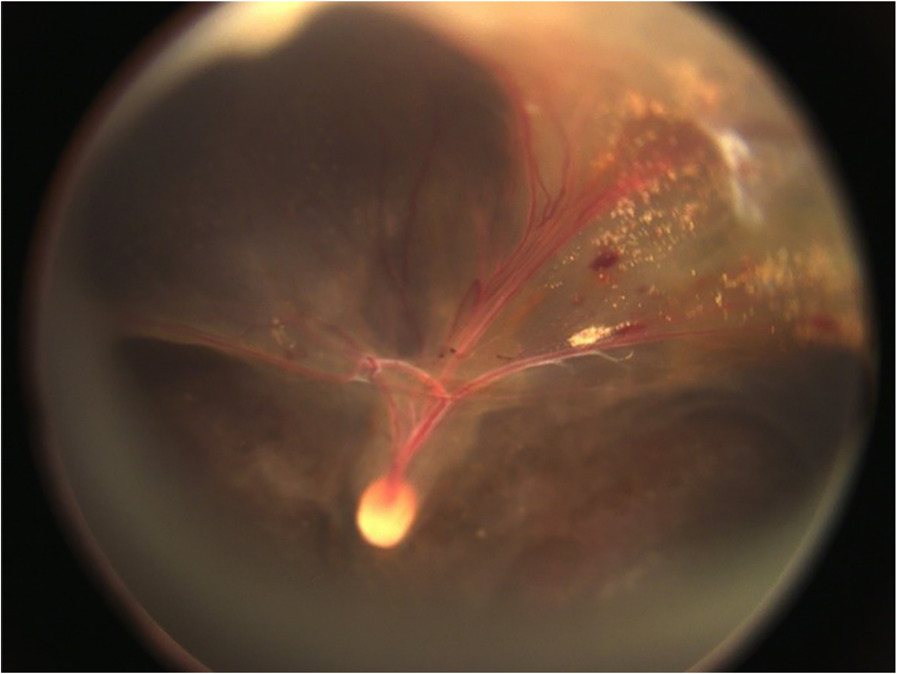
The postoperative fundus image of the left eye in patient no. 17. The radial retinal fold involving all major retinal vessels indicated that the patient might be a FEVR case

Patient no. 17–21 were FEVR patients diagnosed during follow-up postoperatively. Patient no. 17 was misdiagnosed as unilateral PFV preoperatively due to the contralateral eye was completely normal. Considering the shallow anterior chamber, lensectomy was operated to reshape the anterior chamber. During the follow up, the fundus image of the surgical eye showed the radial retinal fold in which all major retinal vessels were involved, which indicating the patient might be FEVR (Fig. 4). Further, we executed FA examination in the patient no. 17 and her parents. Unfortunately, her parents’ FA was positive. The patient’s FA showed that the contralateral eye was completely normal and she was diagnosed as unilateral FEVR.

**Fig. 5:**
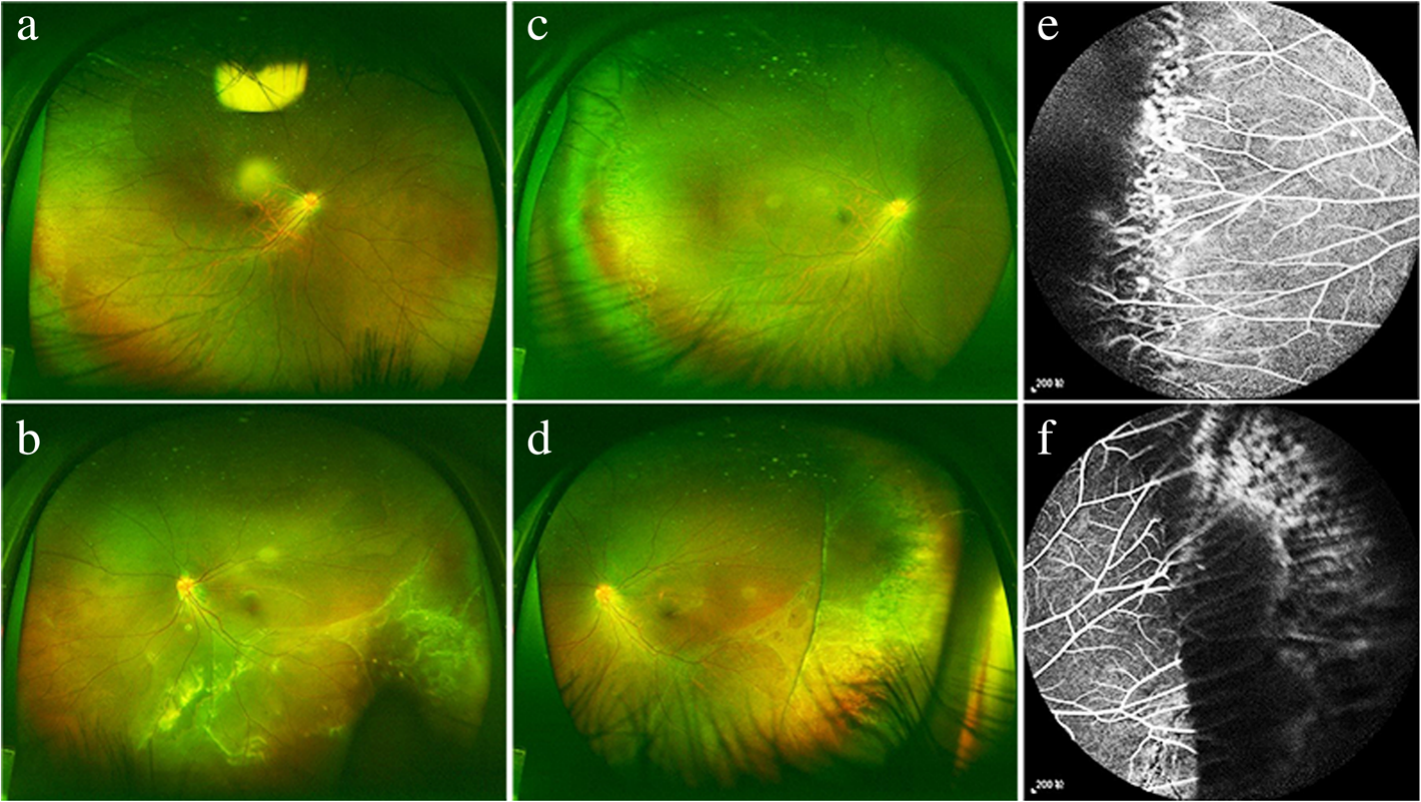
The ultra-widefield fundus image and angiograms of patient no. 18. **a** and **b** The fundus image of the patient first presentation to our clinic. It showed that there were some laser spots in the periphery of the right eye and RD in the temporal and inferior of the left eye. **c** and **d** The fundus image of the patient in 1 month after scleral buckling surgery in our department. The peripheral avascular zones of both eyes were found. **e** and **f** The angiograms of the patient showed plenty of vascular ramifications and laser spots in both eye and impressed ridge in the left eye

Patient no. 18–20 were all complicated with high myopia. The RD in these cases were prone to be diagnosed as non-specific RD. Both eyes of patient no. 18 underwent a photocoagulation therapy in the referral hospital, and the laser scars made FEVR diagnosis more difficult. In our department, scleral buckling was performed in the RRD eye. In a month after the surgery, we found the peripheral avascular area over the scleral indentation zone. Then, FA examination confirmed the diagnosis of FEVR (Fig. 5).

## Discussion

FEVR is a heterogeneous genetic disease. The manifestations of FEVR are greatly variable, which lead to the missed diagnosis of FEVR. Our results showed that FEVR patients who needed surgery might manifest with unilateral RD, bilateral RD, disappearance of anterior chamber, retrolental membrane, ocular trauma, VH, ERM, nystagmus, phthisis bulbi, LMH, MF, HM. All of the ocular presentations were so confusing that missed diagnosis of FEVR was easily made. There were also many case reports about FEVR being mistaken for other vitreoretinopathies, including PFV, VH, ROP, Rb, full-thickness macular hole (FTMH), which strengthened our findings [12–19]. The presentation of FEVR may mimic the presentation of other pediatric and adult vitreoretinopathies, thus careful examination of peripheral retina and avascular peripheral zone is often essential in making the diagnosis of FEVR.

In the study, risk factors analysis showed that the patients who had RD only were more likely to be diagnosed as FEVR preoperatively, while the patients who had other ocular manifestations except RD were more likely to be diagnosed as FEVR intra−/post-operatively. Because the rate of RD in FEVR patients is relatively high. It is reported that RD occurred in 20% eyes with FEVR and 47.6% nonsyndromic congenital RD patients were FEVR patients [7, 20]. In addition, the types of RD in FEVR patients were very variable, such as RRD, falciform retinal folds, PVR with formation of a retrolental membrane and exudative retinal detachment (ERD). And the undetached fellow eyes of FEVR-RRD patients were generally characterized by vascular leakage, lattice degeneration, vitreous traction in the peripheral retina [5]. Considering the high rate of RD in FEVR, when we received a RD patient, we would spontaneously think of the possibility of FEVR. And, carefully examination of peripheral retina in the contralateral eyes of RD patients could be benefit to find FEVR. Bilateral dilated fundoscopy is mandatory and the clear image of peripheral retina is critical. Moreover, parent’s FA is also an important clue which contributes to diagnose FEVR earlier, especially for patients with bilateral disappeared anterior chamber and invisible fundus. In this condition, we should perform FA in his/her parents preoperatively to confirm FEVR. Although the positive family history is not a prerequisite for FEVR, a positive family history is greatly helpful.

In addition, it was noteworthy that there were more patients diagnosed as FEVR preoperatively in recent 3 years than the first 6 years before 2016 (Table 2). First, this was due to the extensive application of ultra-widefield scanning laser ophthalmoscope (UWF SLO) in our department in recent 3 years. UWF SLO could provide a 200° photographic view of a fundus, and it was a valuable imaging tool for detecting fundus anomalies, especially the peripheral retina. It was reported that UWF SLO could assist in the diagnosis and evaluation of early-stage FEVR [21]. In the study, the most common reason for missed diagnosis of FEVR preoperatively was that there was no ultra-wide-field scanning laser ophthalmoscopy at initial clinic visit or preoperatively. (Table 3) Second, over time, our understanding of FEVR was deeper and clinical experience was more abundant, and more and more patients could be accurately diagnosed as FEVR before surgery. The longitudinal changes in the diagnosis and management of FEVR over the years might have introduced bias into risk factor analysis. Thus, in the study, wide-angle fundus imaging preoperatively and year of diagnosis were not independent risk factors of diagnostic timing in the FEVR patients. More cases would be required for further confirmation.

However, when FEVR complicated with other ocular presentations, the diagnosis of FEVR would still be quite challenging. In the study, we concluded procedures for diagnosing FEVR earlier. For patients with unilateral RD only, careful examination of the contralateral eye was essential to find peripheral vascular anomalies especially in adolescent. For patients with bilateral RD only, we should be more alert to the possibility of FEVR and it was necessary to inspect the peripheral retinal vessel with ultra-widefield retinal image. Ultra-widefield fundus photography was important in the management of pediatric retinal diseases and could aid the physician in the documentation and evaluation of peripheral retinal pathology [22]. For pediatric patients with unilateral disappeared anterior chamber, careful examination of peripheral retina of the contralateral eye was critical and parent’s FA could help us to differentiate FEVR with PFV. If parent’s FA is negative, we should perform FA for the pediatric patient who could not cooperate with examination during surgery under general anesthesia. For pediatric patients with bilateral disappeared anterior chamber, we performed FA in their parents. If the parents’ FA were positive, the diagnosis of FEVR could be made preoperatively. In addition, for patients with bilateral ocular fundus disorders except RD without any systemic disorders, we should make the distinction between FEVR and non-specific vitreoretinopathies. During the surgery, observing peripheral retina with simultaneous scleral impression could avoid the missed diagnosis of FEVR, especially for suspected FEVR cases preoperatively. We wished that the procedures would help clinicians diagnose FEVR earlier.

Limitations of the study were as follows: this was a retrospective clinical study; the number of cases was small since FEVR is a relatively uncommon disease; in addition, genetic sequencing was not listed in the study. Because genetic sequencing was generally too expensive to some families in the study. And some parents did not undergo FA examination due to their absence to our clinic or sensitivity to fluorescein sodium. However, not all FEVR patients have FEVR gene mutations and positive family history. Rao FQ et.al reported that 38.7% Chinese patients with FEVR were confirmed to harbor mutations in the six known disease-causing genes [23]. Kashani et.al reported that, 43% FEVR patients had detectable mutations in FZD4, NDP, or TSPAN12 in their study cohort, while only 8% FEVR patients reported a positive family history of FEVR in a first-degree relative [24]. Thus, Genetic sequencing and family history is not a prerequisite for FEVR diagnosis. Namely, the lack of genetic sequencing and family history did not influence our correct diagnosis of FEVR, although a positive family history could contribute to diagnose FEVR earlier. The diagnosis of FEVR was ultimately based on clinical findings.

## Conclusions

In summary, the phenotype of FEVR was greatly variable, they can disguise as many non-specific vitreoretinopathies. The most non-specific referral diagnosis or pre-operative diagnosis was unilateral RD, bilateral RD, unilateral PFV, bilateral PFV. Furthermore, the more complicated the patient’s manifestation, the more delayed the diagnosis of FEVR will be. A positive family history or a simple ocular presentation with RD only could contribute to diagnose FEVR preoperatively. Carefully examination of the contralateral asymptomatic eye and FA in parents preoperatively might be helpful to diagnose complicated FEVR earlier. Patient’s FA during the operation and careful inspection of the peripheral area of retina intraoperatively might prevent missed diagnosis of FEVR in suspected cases.

## Data Availability

The datasets used and/or analyzed during the current study available from the corresponding author on reasonable request.

## Abbreviations

AC: Anterior chamber
ERM: Epiretinal membrane
FEVR: Family exudative vitreoretinopathy
FTMH: Full-thickness macular hole
HM: High myopia
ILM: Internal limiting membrane
L: Lensectomy
LMH: Lamellar macular hole
LV: Lensectomy and vitrectomy
MF: Myopic foveoschisis
PC: Photocoagulation
PFV: Persistent fetal vasculature
RB: Retinoblastoma
RD: Retinal detachment
ROP: Retinopathy of prematurity
SB: Scleral buckling
SiO: Silicone oil
SiOR: Remove silicone oil
V: Vitrectomy
VH: Vitreous hemorrhage
